# Development and Validation of the Chinese Attitudes to Starting Insulin Questionnaire (Ch-ASIQ) for Primary Care Patients with Type 2 Diabetes

**DOI:** 10.1371/journal.pone.0078933

**Published:** 2013-11-13

**Authors:** Sau Nga Fu, Weng Yee Chin, Carlos King Ho Wong, Vincent Tok Fai Yeung, Ming Pong Yiu, Hoi Yee Tsui, Ka Hung Chan

**Affiliations:** 1 Department of Family Medicine and Primary Health Care, Kowloon West Cluster, Hospital Authority, Hong Kong S.A.R., China; 2 Department of Family Medicine and Primary Care, Li Ka Shing Faculty of Medicine, University of Hong Kong, Hong Kong S.A.R., China; 3 Department of Medicine and Geriatrics, Our Lady of Maryknoll Hospital, Hong Kong S.A.R, China; Pennington Biomedical Research Center, United States of America

## Abstract

**Objectives:**

To develop and evaluate the psychometric properties of a Chinese questionnaire which assesses the barriers and enablers to commencing insulin in primary care patients with poorly controlled Type 2 diabetes.

**Research Design and Method:**

Questionnaire items were identified using literature review. Content validation was performed and items were further refined using an expert panel. Following translation, back translation and cognitive debriefing, the translated Chinese questionnaire was piloted on target patients. Exploratory factor analysis and item-scale correlations were performed to test the construct validity of the subscales and items. Internal reliability was tested by Cronbach’s alpha.

**Results:**

Twenty-seven identified items underwent content validation, translation and cognitive debriefing. The translated questionnaire was piloted on 303 insulin naïve (never taken insulin) Type 2 diabetes patients recruited from 10 government-funded primary care clinics across Hong Kong. Sufficient variability in the dataset for factor analysis was confirmed by Bartlett’s Test of Sphericity (P<0.001). Using exploratory factor analysis with varimax rotation, 10 factors were generated onto which 26 items loaded with loading scores > 0.4 and Eigenvalues >1. Total variance for the 10 factors was 66.22%. Kaiser-Meyer-Olkin measure was 0.725. Cronbach’s alpha coefficients for the first four factors were ≥0.6 identifying four sub-scales to which 13 items correlated. Remaining sub-scales and items with poor internal reliability were deleted. The final 13-item instrument had a four scale structure addressing: ‘Self-image and stigmatization’; ‘Factors promoting self-efficacy; ‘Fear of pain or needles’; and ‘Time and family support’.

**Conclusion:**

The Chinese Attitudes to Starting Insulin Questionnaire (Ch-ASIQ) appears to be a reliable and valid measure for assessing barriers to starting insulin. This short instrument is easy to administer and may be used by healthcare providers and researchers as an assessment tool for Chinese diabetic primary care patients, including the elderly, who are unwilling to start insulin.

## Introduction

The global burden of diabetes mellitus is rapidly increasing and it is estimated that worldwide, over 285 million adults now suffer from Type 2 diabetes mellitus (T2DM) [1]. T2DM is a metabolic condition characterised by insulin resistance causing reduced responsiveness to the effect of insulin on peripheral tissues, resulting in high blood sugar levels. Insufficient insulin secretion to overcome insulin resistance is also a feature of the condition. T2DM has become a major public health problem in the Chinese, with prevalence rates in China rising sharply in the past decade to approximately 9.7% (accounting for approximately 92.4 million adults) [2]. Located on the Pearl River Delta, Hong Kong is a Special Administrative Region of the People’s Republic of China, with a population which is over 95% ethnically Chinese. Prevalence estimates for T2DM in Hong Kong adults range from 2% in people aged < 35 years to over 20% in those > 65 years [3], [4].

A significant proportion of T2DM is managed in primary care. Hong Kong has a pluralistic health care economy and primary care is provided by both private and public healthcare providers. Government-funded general out-patient clinics provide approximately 15% of all primary care consultations in Hong Kong, focussing mainly on servicing the elderly and those with chronic disease such as diabetes [5].

Several large studies, including the United Kingdom Prospective Diabetes Study (UKPDS), have demonstrated a strong correlation over time between blood glucose control and development of diabetic complications such as kidney failure, blindness, leg amputations, cardiovascular diseases and stroke in patients with T2DM [6]–[8]. Unfortunately, glycaemic control for many T2DM patients worldwide remains sub-optimal which predisposes them to a higher risk of complications and poor health outcomes [9], [10]. Many patients with T2DM are treated with oral medications to help control blood glucose levels. These are taken either alone or in combination, and work by correcting one or more of the metabolic abnormalities which characterise the disease (insulin deficiency, insulin resistance and increased hepatic glucose output) [11]. Monitoring of blood glucose control is usually performed by measuring levels of Haemoglobin A1C (HbA1C) with levels > 7.0 indicating poor control [12]. Better blood glucose control is usually achieved by ‘stepping up’ anti-diabetic treatments through increasing oral therapy, or commencing insulin [12]. Due to the progressive nature of T2DM, insulin therapy is eventually indicated for many patients once maximal doses of oral medications are no longer sufficient to control blood sugar levels (‘failed oral therapy') [12].

Notwithstanding that insulin is a safe and effective drug for achieving glycaemic control [13], [14], it is a global phenomenon that most T2DM patients resist starting insulin, predominantly because of psychological reasons (termed ‘psychological insulin resistance’) [15]–[22]. The decision to start insulin is often difficult and patients’ reluctance may cause delays in initiating therapy, prolonging their sub-optimal glycaemic control [19]. Unwillingness or refusal to start insulin has been found to be more common in Chinese patients. Studies conducted in Chinese populations report over 70% of T2DM patients are unwilling to start insulin [23], which is higher than in non-Chinese patients where reported resistance or refusal rates have ranged from 28.2% to 46.6% [20], [24], [25].

Reluctance to commence insulin may be a result of a range of personal viewpoints involving cognitive appraisal or emotional reactions [26], which can be influenced by culture [27]–[29], degree of self-efficacy and health literacy [30]. Chinese patients appear to be more concerned about the psycho-social aspects of insulin treatment such as impact on self-image, social stigmatization, or inability to acquire the necessary skills, than the physical aspects such as having a hypoglycaemic attack or weight gain [26], [27], [31].

A number of questionnaires have been developed which assess patient attitudes towards insulin therapy [32]–[34] including the Chinese version Insulin Treatment Appraisal Scale (ITAS) [31], however none have been designed or validated for use in predominantly elderly primary care patients who have not yet started insulin therapy (referred to as ‘insulin naïve’ patients). Having an instrument which easily identifies the patients’ reasons for refusing insulin would be a valuable assessment tool for healthcare providers enabling them to more effectively tailor educational interventions to help overcome their concerns.

As there was no suitable assessment tool available, the aim of this study was to develop and validate a Chinese questionnaire which assesses the barriers and enablers to starting insulin treatment in insulin naïve T2DM patients with the following objectives:

1. To identify relevant items which can be used to assess patient attitudes regarding starting insulin
2. To translate the items into Chinese.
3. To pilot the developed instrument on a primary care population to assess acceptability and feasibility of administering the questionnaire to elderly patients with T2DM.
4. To assess the psychometric properties of the translated instrument.

## Methods

The Research Ethics Committee of the Kowloon West Cluster, Hospital Authority of Hong Kong granted research ethics approval of the research protocol.

### Instrument Development

Twenty-seven potential items were originally identified. Twenty-six were derived from literature review (Table 1) with a further one item derived from a pilot study conducted on local T2DM patients [35]. A six-person expert panel of health care providers (comprised of 4 primary care doctors, 1 endocrinologist and 1 nurse specialized in diabetes care) were invited to review the items for content, breadth, and relevancy and to rate each item on validity, relevance. A content validity index (CVI) was calculated for each item. Items scoring ≥ 80% were retained [36]. Items with CVI <80% were eliminated or revised. The items were then formatted to create a structured English questionnaire with a four-point Likert scale response option for each item (from Strongly Agree to Strongly Disagree).

**Table 1:** Item Identification and Fictitious Ratings on relevance of 27-Item Scale by Experts.

Questionnaire Items from literature review [15], [17], [18], [20]–[23], [27],	Reference Number		Rating on Relevance* by Expert					Experts in Agreement	Item CVI
			1	2	3	4	5		
Concern of Injection									
Pain	[17], [18], [20]–[23], [27], [30], [32]–[35], [38]		✓	✓	-	✓	✓	4	80%
Cannot manage skill of injection	[17], [18], [20], [21], [23], [27], [30], [34], [35], [38]		✓	✓	✓	✓	✓	5	100%
Inconvenience	[17], [20], [21], [33], [35], [38]		✓	✓	✓	✓	✓	5	100%
Complication of injection (skin marking and others)	[20], [33]–[35], [38]		✓	✓	✓	✓	✓	5	100%
Afraid of injection	[20], [22], [32]–[34], [38]		✓	✓	✓	✓	✓	5	100%
Afraid of blood glucose monitoring	[30], [32]		✓	✓	✓	✓	✓	5	100%
Concern of cost									
Financial cost	[17], [20], [22], [27], [30], [35]		✓	✓	✓	✓	✓	5	100%
Time (for regular injection)	[20], [21], [27], [32]–[35], [38], [39]		✓	✓	✓	✓	✓	5	100%
Concern of social/family									
Affect social life	[18], [20], [23], [27], [30], [32]–[34], [38], [39]		✓	✓	✓	✓	✓	5	100%
Worry other people will know his/her problem	[17], [23], [30], [33]–[35], [39]		✓	✓	✓	✓	✓	5	100%
Feeling injection is embarrassing, afraid of being seen	[15], [17], [20], [27], [32]–[34]		✓	✓	✓	✓	✓	5	100%
Feels like drug addict	[32]		✓	✓	✓	✓	✓	5	100%
Lack of family support	[15], [34], [35], [38]		✓	✓	✓	✓	✓	5	100%
Lack of social support	[20], [21], [38]		✓	✓	✓	✓	✓	5	100%
Concern of the DM disease management									
Lack of updated information about DM	[15], [22], [38]		✓	✓	✓	✓	✓	5	100%
Low trust to physician	[20], [30], [33]		✓	✓	✓	✓	✓	5	100%
Wish to try Traditional Chinese Medicine	[35]		✓	✓	✓	✓	✓	5	100%
Wish to try other method (life style or alternative medicine) to control DM	[20]		✓	✓	✓	✓	-	4	80%
Worry about dependence on the drug	[32], [35]		✓	✓	✓	✓	✓	5	100%
Insulin will worsen disease severity	[23], [32]–[35]		✓	✓	✓	✓	✓	5	100%
Do not believe insulin can help his/her disease (glucose control and prevent complication)	[17], [18], [20], [23], [30], [32], [34], [35], [39]		✓	✓	✓	✓	-	4	80%
Pills works better than insulin	[17], [32]		✓	✓	✓	✓	-	4	80%
Do not agree that his/her disease is severe	[17], [18], [20], [33]–[35], [38], [39]		✓	✓	✓	✓	✓	5	100%
Insulin injection means failure of treatment	[15], [21], [34], [39]		✓	✓	✓	✓	-	4	80%
Can’t pay close attention to my diet as insulin treatment requires	[20], [32]–[34]		✓	✓	✓	✓	✓	5	100%
Worry about hypoglycemia	[17], [18], [20], [21], [23], [27], [30], [32]–[34]		✓	✓	✓	✓	-	4	80%
Worry about weight gain	[22], [23], [34]		✓	✓	✓	✓	✓	5	100%
Average CVI									96%
Proportion relevant (%)		100		100	96	100	81		

The questionnaire was translated into Chinese by the principal investigator (SF) and translated back into English by another co-investigator (MY) to assess translational equivalence. Discrepancies between the original English items and back-translated items were reviewed by both investigators. All nonequivalent items were modified to enhance their translational equivalence to the original English version. Both investigators are bilingual with previous experience in translation of questionnaire surveys. The resulting Chinese instrument underwent field testing and cognitive debriefing interviews using 10 patients with different distributions of age, sex, and previous insulin use.

### Pilot psychometric testing of the Ch-ASIQ

The 27 item Chinese Attitudes to Starting Insulin Questionnaire (Ch-ASIQ) was pilot-tested on primary care patients recruited from ten Hospital Authority primary care clinics across Hong Kong. All eligible patients attending any of the study locations during the study period were invited to participate. Eligible subjects were identified through the Hospital Authority’s computer dispensing system and invited to complete the questionnaire when they attended the clinic for a scheduled follow-up appointment. As a large proportion of patients attending these clinics are elderly with low literacy levels, trained research assistants helped to explain the study, obtain signed consent and administered the questionnaires.

All eligible subjects were consecutively recruited until the required sample size was reached. Sample size calculation was based on the number needed to perform the factor analysis for psychometric assessment of the instrument. As there were 27 potential items, based on the subject to item ratio of 10:1 [37], a sample size of 270 subjects was required. Inclusion criteria were: Chinese-speaking adults aged ≥18 or ≤80; on maximum recommended or maximum tolerable doses of oral diabetic medications (Gliclazide 320 mg, Gliclazide modified release 120 mg, or Glibenclamide 15 mg and metformin ≥2 g daily) ; most recent HbA1c level ≥7.5% within past 12 months indicating insufficient glycaemic control [6], [12]. Exclusion criteria included: pregnancy; unable to answer a questionnaire due to mental incapacity; or already on insulin therapy.

### Statistical Analysis

Descriptive statistics were calculated with median and inter-quartile ranges (IR) for continuous variables, and frequency and proportion for categorical variables. Negative items were re-coded and responses scored from one to four with higher scores indicating more positive attitudes. Exploratory factor analysis (EFA) was used to explore the underlying structure of the instrument and to sort items into sub-scales. A factor loading score ≥0.4 was used to sort items into factors. Items which cross-loaded across two factors, and one-item factors were deleted. The Kaiser–Meyer–Olkin (KMO) measure of sampling adequacy (using a cut-off of 0.5), and Barlett’s Test of Sphericity (using a cut-off P<0.001) was used to ensure the appropriateness of the data set for EFA. Cronbach’s alpha coefficient was used to assess the internal reliability of each sub-scale identified by EFA. A Cronbach’s alpha coefficient ≥ 0.6 was used as the cut-off to indicate sufficient internal reliability [37].

IBM SPSS Statistics for Windows, Version 20.0 statistical software was used to conduct descriptive and exploratory factor analyses.

## Results

Twenty-seven items (Table 1) underwent content validation. The calculated CVI of all items scored > 80% for all items and were retained. All items on cognitive debriefing also yielded scores > 80%. (Table 1)

306 eligible subjects were approached and 303 subjects completed the questionnaire (response rate  = 99%). The socio-demographic and clinical characteristics of the subjects are shown in Table 2. Typical of the patient population attending government-funded primary care clinics, subjects were elderly, had lower levels of education, and only one third were in full-time employment. The median duration of T2DM was 11 years (IR  =  7 to 16 years) and median HbA1c level was HbA1c level 8.3% (IR  =  7.9 to 9.1%) indicating very poor levels of glycaemic control.

**Table 2:** Sociodemographic and Clinical Characteristics of Type 2 Diabetes Patients at Baseline.

Characteristics		Total (N = 303)
***Sociodemographic***		
Age (median year, IQR)		63 (54–70)
Gender (%)		
	Male	136 (44.9%)
	Female	167 (55.1%)
Education (%)		
	No formal education	44 (15.4%)
	Primary	117 (41.1%)
	Secondary	107 (37.5%)
	Tertiary	17 (6.0%)
Occupation (%)		
	Full time work	90 (32.8%)
	Unemployed/retired	82 (29.9%)
	Housewife	99 (36.1%)
	Part time	3 (1.1%)
Mode of Administration (%)		
	Self	104 (34.4%)
	Interviewer	189 (62.6%)
		
***Clinical***		
Duration of DM (median year, IQR)		11 (7–16)
Last HbA1c Level (median %, IQR)		8.3 (7.9–9.1)
Hypertension (%)		246 (81.2%)
DM drug (%)		
	Glibenclamide	37 (12.2%)
	Gliclazide	259 (85.5%)
	Metformin	303 (100.0%)

Note: IQR, Interquartile range.

Using the principal component EFA with varimax rotation, ten factors with eigenvalues ≥1 were extracted as shown in Table 3. The KMO measure was 0.725 indicating sampling adequacy. Sufficient variability in the data was confirmed by Bartlett’s Test of Sphericity (P<0.001) confirming the validity of data available for EFA. The ten factors, onto which 26 items with the absolute magnitude of factor loadings exceeded 0.4, explained 66.22% of the total variation. Item 7 was itself regarded as a one-item factor, and was dropped for subsequent analysis. No items cross-loaded over more than one factor.

**Table 3:** Factor loadings for 27-item Barrier to Insulin Questionnaire.

	Rotated Factor loading (N = 247)										
Item #	Factor 1	Factor 2	Factor 3	Factor 4	Factor 5	Factor 6	Factor 7	Factor 8	Factor 9	Factor 10	
1. Lack of updated information about DM	0.075	0.701	0.035	–0.352	–0.176	0.177	0.052	–0.084	0.267	–0.021	
2. Low trust to physician	0.118	0.137	0.095	–0.040	0.006	–0.110	0.815	0.064	–0.159	0.104	
3. Wish to trying Traditional Chinese Medicine	0.224	–0.003	–0.080	–0.101	0.030	–0.153	0.012	0.092	0.812	–0.004	
4. Wish to try lifestyle (diet control and exercise) or other alternative medicine	–0.165	–0.143	0.008	0.098	0.099	0.302	0.669	0.008	0.322	–0.077	
5. Feelings of drug dependence	0.156	0.198	–0.026	0.008	0.234	0.042	0.048	0.689	0.051	0.020	
6. Insulin will worsen disease severity	0.081	–0.011	0.073	–0.170	0.715	0.149	–0.011	0.202	0.093	0.132	
7. Do not agree that his/her disease is severe	–0.151	0.145	0.178	–0.019	–0.117	0.156	0.063	0.356	0.161	0.605	
8. Do not believe insulin can help control blood glucose and prevent complications	–0.010	0.539	–0.124	0.319	–0.251	0.115	0.058	0.086	–0.126	0.076	
9.Pills work better than insulin	0.018	–0.145	0.124	–0.157	0.540	0.146	0.270	–0.018	–0.379	–0.265	
10. Means failure of the diabetes treatment	0.051	–0.128	0.136	0.344	0.442	0.387	–0.125	0.029	0.150	–0.037	
11. Fear of hypoglycemia	0.205	–0.138	0.093	–0.043	0.141	0.731	0.009	0.217	–0.022	0.044	
12. Fear of weight gain	0.209	0.117	–0.040	–0.059	0.099	0.734	0.064	–0.157	–0.227	0.043	
13. Pain	0.001	0.022	0.648	0.059	0.290	0.357	0.098	–0.248	0.129	0.043	
14. Cannot manage skill of injection	–0.125	0.593	–0.141	0.388	–0.105	–0.088	–0.008	–0.128	0.098	0.226	
15. Inconvenience	0.187	–0.171	0.348	0.083	0.476	0.048	0.299	–0.121	–0.089	–0.146	
16. Afraid of injection	0.180	–0.088	0.826	–0.161	0.074	–0.033	0.142	–0.071	–0.017	0.036	
17. Afraid of blood glucose monitoring	0.101	–0.146	0.673	–0.034	0.025	–0.037	–0.104	0.230	–0.158	–0.105	
18. Complication of injection (skin mark & others)	0.385	–0.150	0.344	–0.311	–0.100	0.315	0.150	0.026	0.042	0.031	
19. Financial cost	0.149	0.064	–0.128	0.065	0.073	–0.010	0.009	–0.128	–0.081	0.825	
20. Time (for regular dose of insulin)	–0.099	0.065	–0.046	0.754	–0.101	0.067	0.098	0.162	–0.130	0.077	
21. Affect social life or hobbies	0.394	0.086	–0.004	–0.265	0.421	0.038	–0.032	–0.517	–0.128	0.048	
22.Worry other people will know his/her problem	0.816	0.054	0.087	–0.141	0.014	0.097	–0.084	0.058	0.105	0.071	
23.Feeling injection is embarrassing, afraid of being seen	0.860	0.027	0.150	–0.050	–0.021	0.141	0.037	–0.068	0.037	–0.057	
24. Feels like drug addict	0.736	–0.098	0.048	–0.026	0.229	0.107	0.066	0.083	0.064	0.040	
25. Lack of family support	–0.136	0.260	–0.089	0.723	–0.024	–0.138	–0.061	–0.102	0.048	–0.032	
26.Lack of social support	–0.008	0.696	–0.117	0.100	–0.042	–0.154	0.035	0.196	–0.147	0.043	
27.Can't pay close attention to my diet as insulin treatment requires	0.006	0.616	–0.036	0.179	0.379	–0.072	–0.103	0.097	0.035	0.032	
											Total
Eigenvalue	4.606	2.613	2.100	1.494	1.378	1.271	1.233	1.119	1.059	1.007	17.87
% of variance	0.171	0.097	0.078	0.055	0.051	0.047	0.046	0.041	0.039	0.037	0.662

Note:

Factors were extracted by exploratory factor (principal component) analysis with varimax rotation.

The Kaiser–Meyer–Olkin (KMO) measure was 0.725.

For ease of clinical interpretation, the remaining nine factors (excluding the tenth factor with eigenvalue marginally greater than one) were collapsed to seven sub-scales. The descriptive scores and the proportion of subjects rating ‘agree’ or ‘strongly agree’ for each item according to sub-scale categorisation, with the Cronbach’s alpha coefficient of each sub- scale is shown in Table 4. Sub-scale (6) interpreted as ‘Worry about complications of insulin therapy’ was the combination of factor 6 and factor 8, whereas sub-scale (7), interpreted as ‘Trust in health professionals’, was the combination of factor 7 and factor 9. Internal consistency of the seven sub-scales was assessed using the Cronbach’s alpha. Four of the sub-scales had Cronbach’s alpha values >0.6 indicating sufficient internal consistency. The remaining sub-scales had poor internal consistency and those items were removed.

**Table 4:** Mean scores and distribution of responses to individual items, and Internal consistency for each scale.

Scale/individual item		Mean±SD	Agree/Totally Agree (%)	Cronbach's alpha if item deleted
Scale 1: Self image and stigmatization (Cronbach's alpha = 0.802)				
Item #22	I worry that people will know I have diabetes if I am on insulin treatment	2.36±0.85	121 (40.33%)	0.702
Item #23	Injecting insulin is embarrassing, I worry about being seen when I inject insulin	2.49±0.81	147 (49.16%)	0.663
Item #24	If I have to inject insulin, it makes me feel like drug addicts	2.45±0.80	133 (44.93%)	0.812
Scale 2: Factors promoting self-efficacy (Cronbach's alpha = 0.675)				
Item #01	I have up-to date knowledge about diabetes management	2.60±0.83	189 (63.21%)	0.670
Item #08	Insulin can help control blood glucose and prevent complications	2.65±0.68	181 (63.73%)	0.618
Item #14	I can manage the skill of injecting insulin	2.72±0.75	201 (67.45%)	0.592
Item #26	There is social support available if I have to inject insulin	2.37±0.68	131 (44.41%)	0.601
Item #27	I can pay as close attention to my diet as insulin treatment requires. For example, I may need to take snack or reduce eating amount appropriately according	2.67±0.66	196 (66.67%)	0.640
Scale 3: Fear of pain or needles (Cronbach's alpha = 0.653)				
Item #13	Injecting insulin is painful	2.79±0.73	200 (66.89%)	0.620
Item #16	I am afraid of needle injections	2.91±0.86	211 (70.57%)	0.340
Item #17	I worry about needing to perform home blood sugar monitoring	2.59±0.82	158 (53.02%)	0.656
Scale 4: Time & family support(Cronbach's alpha = 0.620)				
Item #20	I can spare enough time to perform insulin injection	2.58±0.71	176 (59.46%)	NA
Item #25	My family will support me to inject insulin	2.45±0.73	133 (45.70%)	NA
Scale 5: Misunderstanding of insulin therapy (Cronbach's alpha = 0.573)				
Item #06	Insulin can cause permanent damage or worsening of my health.	2.50±0.71	133 (45.86%)	0.512
Item #09	Diabetes tablets work better than insulin	2.82±0.70	205 (69.26%)	0.460
Item #10	Insulin injection means failure of the diabetes tablet treatment	2.73±0.64	197 (66.55%)	0.560
Item #15	Injecting insulin is inconvenient	3.05±0.72	242 (80.40%)	0.463
Scale 6: Worry about complications of insulin therapy (Cronbach's alpha = 0.488)				
Item #05	Insulin treatment for diabetes causes feelings of drug dependence.	2.63±0.69	172 (58.70%)	0.550
Item #11	An insulin overdose can lead to extremely low blood-sugar levels ("hypoglycemia"). I am afraid of experiencing the symptoms of low blood sugar levels	2.61±0.68	168 (57.53%)	0.337
Item #12	I worry about weight gain associated with insulin injections	2.40±0.65	117 (39.80%)	0.374
Item #18	I worry about skin marks or skin complications associated with injecting insulin	2.56±0.72	152 (51.18%)	0.383
Item #21	Insulin treatment will make life less flexible, affect my social life and hobbies (e.g. performing exercise, dinning outside)	2.67±0.73	174 (58.78%)	0.485
Scale 7: Trust in health care professionals (Cronbach's alpha = 0.203)				
Item #02	I trust that my doctor is providing me with the most appropriate diabetes management for me	3.22±0.60	278 (92.67%)	NA
Item #03	I wish to or I am now trying Traditional Chinese Medicine to control blood sugar	2.35±0.79	130 (43.05%)	NA
Item #04	I wish to or am now trying lifestyle (diet control and exercise) or other alternative medicine (e.g. complementary medicine, Qi Kung, etc) to control blood sugar	3.15±0.58	280 (92.72%)	NA

Note:

NA = Not applicable due to small number of items.

The final instrument yielded 13 items with four sub-scales (Appendix S1 and Appendix S2(Chinese Version)) which were interpreted as (1) ‘Self-image and stigmatization’; (2) ‘Factors promoting self-efficacy’ (3) ‘Fear of pain or needles’; and (4) ‘Time & family support’.

## Discussion

This is the first report describing the development and psychometric validation of a Chinese questionnaire that assesses barriers and enablers to starting insulin therapy in insulin naïve T2DM primary care patients. The instrument is based on translations and adaptation of six different questionnaires [19], [32]–[34], [38], [39] and literature reviews (Table 1) and has undergone assessment for translational equivalence, and content validation to ensure items are appropriate for application to Chinese primary care patients. The questionnaire was able to be understood by both males and females of varying ages including elderly patients and those with lower educational levels. The questionnaire was understood by patients who had and had not previously used insulin.

Consistent with other psychometric validated questionnaires [32]–[34], the Ch-ASIQ contained two subscales which measured two common psychological barriers to insulin treatment: stigma of insulin use and fear of injections. Insulin therapy is commonly associated with negative connotations and often causes dysfunctional emotions such as fear, anxiety [26]. In clinical practice clinicians need to take into consideration their patients’ negative emotions and concerns when they counsel patients about the need to start insulin. It is therefore appropriate that items addressing these issues should be included in a clinical assessment tool. Similarly, the Ch-ASIQ contained two subscales which measured the patient’s perceived needs in terms of personal resources required to take on the added responsibility of insulin therapy. In clinical practice, it is also important to identify ways to empower patients so that they can better look after their health and an assessment of needs in terms of knowledge, skills, social support and time should be factored into an evaluation of an individual’s readiness to adhere to any changes in drug regimen [40].

Although components of social and family support are rarely mentioned in other questionnaires, it appears to be quite important for Chinese patient populations. Family engagement is important in Chinese culture and there appears to be a correlation between the amount of perceived family support and health behaviors in Chinese patients with chronic diseases, in particular, those who are elderly [26], [35], [38], [41]. Such support is also important for patients with lower education levels and lower health literacy as they may require additional assistance to follow the instructions of a new prescription [30].

Time appears to be an important factor in our setting. Despite the fact that less than one third of the tested subjects were in full-time employment, time was still considered consistently an essential item in the subscale relating to personal resources. This likely reflects the culture of Hong Kong society as its citizens live in one of the fastest paced countries in the world [42], have long working hours, and have limited free time [43].

The deleted items from the sub-scales with low Cronbach’s alpha coefficients reflect values and attitudes which are less significant and less consistently considered in our setting. Items within the sub-scales interpreted as ‘Misunderstanding of insulin therapy’; ‘Worry about complication of insulin therapy’; ‘Trust in health care professionals’ appear to be less important in our study population possibly due to their lower levels of education, age, and ethnicity. Chinese elderly patients appear to be less likely to question the doctor’s expertise or advice [44].

The items related to fear of hypoglycemia, weight gain and complications of insulin which appear to be important in other studies [17], [18], [20], [21], [23], [27], [30], [32]–[34] were not consistently weighted in the exploratory factor analysis of this study population. Similar findings were also found in another study interviewing Chinese subjects [27]. One explanation is that the anxiety evoked by injections far exceeds the anxiety evoked by any other factor.

Other studies have hypothesized that one reason for insulin refusal is that Chinese patients might not trust Western Medicine [27]. However, in this study, the items related to distrust of Western medicine were also deleted, reflecting that these were not major and consistent barriers among our target subjects.

There were a number of limitations to this study. The questionnaires were interviewer-administered in majority of the subjects as our patient population has poor literacy levels. It is possible that the Ch-ASIQ’s psychometrics may differ if self-administered. We chose to keep only those items that demonstrated a clear and unambiguous factor loading and some of the items that were excluded after factor analyses may still be relevant for patients in other settings. Test-retest reliability was not been performed and further studies to examine the responsiveness of the instrument (ability to detect change) following intervention or over time are still required.

## Conclusion

The 13-item Chinese Attitudes to Starting Insulin Questionnaire (Ch-ASIQ) offers reliable psychometric properties as well as an interpretable and relevant structure. Our findings suggest that the Ch-ASIQ can be used by clinicians and researchers in a valid and reliable way to assess and address psychological barriers to insulin treatment in Chinese T2DM subjects in primary care setting. The future application of this instrument will be to guide the development of tailored education interventions to help these patients accept and initiate insulin therapy, and to assess the outcomes of the interventions.

## Supporting Information

Appendix S1 **The Chinese Attitudes to Starting Insulin Questionnaire (Ch-ASIQ).**
(DOCX)

Appendix S2 **The Chinese Attitudes to Starting Insulin Questionnaire (Ch-ASIQ) (Chinese version).**
(DOCX)
