# Supplementary material for: Development and Validation of the Chinese Attitudes to Starting Insulin Questionnaire (Ch-ASIQ) for Primary Care Patients with Type 2 Diabetes
Source: PLoS One. 2013 Nov 13;8(11):e78933. doi: 10.1371/journal.pone.0078933 (PMC3827341; doi:10.1371/journal.pone.0078933)
Supplement: Appendix S1 — The Chinese Attitudes to Starting Insulin Questionnaire (Ch-ASIQ). (DOCX) [file pone.0078933.s001.docx]

| Appendix S1: The Chinese Attitudes to Starting Insulin Questionnaire (Ch-ASIQ) | | | | | | |
| --- | --- | --- | --- | --- | --- | --- |
|  | Item | English Version | Totally disagree | Disagree | Agree | Totally Agree |
| (a) Self-image and stigmatization | | | | | | |
|  | 1 | I worry that people will know I have diabetes if I am on insulin treatment | o | o | o | o |
|  | 2 | Injecting insulin is embarrassing, I worry about being seen when I inject insulin | o | o | o | o |
|  | 3 | If I have to inject insulin, it makes me feel like a drug addict | o | o | o | o |
| (b) Factors promoting self-efficacy | | | | | | |
|  | 4 | I have up-to date knowledge about diabetes management | o | o | o | o |
|  | 5 | Insulin can help control blood glucose and prevent complications | o | o | o | o |
|  | 6 | I can manage the skill of injecting insulin | o | o | o | o |
|  | 7 | There is social support available if I have to inject insulin | o | o | o | o |
|  | 8 | I can pay as close attention to my diet as my insulin treatment requires. For example, I may need to take a snack or reduce my eating amount appropriately | o | o | o | o |
| (c) Fear of pain or needles | | | | | | |
|  | 9 | Injecting insulin is painful | o | o | o | o |
|  | 10 | I am afraid of needle injections | o | o | o | o |
|  | 11 | I worry about needing to perform home blood sugar monitoring | o | o | o | o |
| (d) Time & Family Support | | | | | | |
|  | 12 | I can spare enough time to perform insulin injections | o | o | o | o |
|  | 13 | My family will support me to inject insulin | o | o | o | o |
