# Supplementary material for: Development and Validation of the Chinese Attitudes to Starting Insulin Questionnaire (Ch-ASIQ) for Primary Care Patients with Type 2 Diabetes
Source: PLoS One. 2013 Nov 13;8(11):e78933. doi: 10.1371/journal.pone.0078933 (PMC3827341; doi:10.1371/journal.pone.0078933)
Supplement: Appendix S2 — The Chinese Attitudes to Starting Insulin Questionnaire (Ch-ASIQ) (Chinese version). (DOCX) [file pone.0078933.s002.docx]

| Appendix S2: 中文版始用胰島素態度問卷 | | |  |  |  |  |
| --- | --- | --- | --- | --- | --- | --- |
|  | Item | Chinese Version | 完全不同意 | 不同意 | 同意 | 完全同意 |
| (a) 自我形象及歧視 | | | | | | |
|  | 1 | 如我注射胰島素，我擔心其他人會知道我有糖尿病 | o | o | o | o |
|  | 2 | 注射胰島素令我覺得尷尬，我擔心注射胰島素時被人看見 | o | o | o | o |
|  | 3 | 如我注射胰島素，我感覺自己像是吸毒者。 | o | o | o | o |
| (b) 提升自我效能感的因素 | | | | | | |
|  | 4 | 我知道糖尿病的最新資訊，例如最新治療的建議及藥物等 | o | o | o | o |
|  | 5 | 注射胰島素可幫助控制血糖及預防併發症(例如中風，心臟病，糖尿上眼，腎衰竭，腳皮膚潰瘍等) | o | o | o | o |
|  | 6 | 如果有醫護人員的協助，我估計我能掌握注射胰島的技巧 | o | o | o | o |
|  | 7 | 我認為有社區資源(例如糖尿病協會)去協助我注射胰島素 | o | o | o | o |
|  | 8 | 因為注射胰島素, 我會或我可以加強飲食控制, 注意自己是否需要適當地減少或額外進食。 | o | o | o | o |
| (c) 害怕針及注射痛楚 | | | | | | |
|  | 9 | 注射胰島素會有痛楚 | o | o | o | o |
|  | 10 | 我害怕用針注射 | o | o | o | o |
|  | 11 | 我擔心要自己在家監察血糖（篤手指） | o | o | o | o |
| (d) 時間的調適及家人支持 | | | | | | |
|  | 12 | 我可安排足夠的時間去注射胰島素 | o | o | o | o |
|  | 13 | 我的家人支持我注射胰島素 | o | o | o | o |
